# Editorial Comment from Dr Kobayashi to Pathological complete response after nivolumab therapy following angiogenesis inhibitors in a patient with metastatic renal cell carcinoma

**DOI:** 10.1002/iju5.12225

**Published:** 2020-10-05

**Authors:** Takashi Kobayashi

**Affiliations:** ^1^ Department of Urology Kyoto University Graduate School of Medicine Kyoto Japan

Little has been reported regarding pathological findings on renal cell carcinomas (RCC) that have shown radiological response to nivolumab treatment. Hagimoto *et al*. reported a case of metastatic RCC (mRCC) who achieved disease‐free status by nivolumab and surgical removal of the residual lesion.^1^ Of note, nivolumab achieved radiological partial response (PR) and pathological complete response (CR) in the right adrenal metastasis, which was progressive after multiple drug regimens including interferon and kinase inhibitors. According to the authors only four cases have been reported as RCC cases achieving pathological CR by nivolumab.

According to updated follow up data,^2^ only 4 (1%) of the 410 patients of the nivolumab arm in CheckMate 025 trial achieved radiological CR, while 90 (22%) showed radiological PR. Of the 90, 31 (34%) was reported to remain off treatment. Additionally, in 65 patients who initially assigned to the everolimus arm and subsequently received the crossover nivolumab treatment, five achieved radiological response (one CR and four PRs) and two of them remained off treatment. Although a substantial part of those achieving durable, off‐treatment disease control may have no residual viable cells, but the frequency is unknown due to low rate of surgical removal of those lesions. Moreover whether or not surgical resection is beneficial for patients who achieved radiological response is a matter of different dimension.

We will have an increasing number of cases of mRCC with durable radiological response to systemic immunological treatment since we have currently more intensified treatment for mRCC including nivolumab plus ipilimumab, avelumab plus axitinib, and pembrolizumab axtinib. Ideally, metastasectomy with curable intent should be considered for those with originally oligometastatic (low metastatic burden) disease when the surgery is technically feasible and expectedly achieves radiologically disease‐free status.^3^ Accumulation of long‐term follow‐up data with or without metastasectomy after radiological response by systemic immunological treatment will provide useful information for the indication of surgical removal of residual lesions in the future.

## Conflict of interest

The author declares no conflict of interest.

## References

[1] Hagimoto H , Kashima S , Doi K *et al* Pathological complete response after nivolumab therapy following angiogenesis inhibitors in a patient with metastatic renal cell carcinoma. IJU Case Rep. 2020; 3: 287–90. 10.1002/iju5.12220 PMC7609183 33163928

[2] Motzer RJ , Escudier B , George S *et al* Nivolumab versus everolimus in patients with advanced renal cell carcinoma: updated results with long‐term follow‐up of the randomized, open‐label, phase 3 CheckMate 025 trial. Cancer 2020; 126: 4156–67. 10.1002/cncr.33033 PMC8415096 32673417

[3] Lyon TD , Thompson RH , Shah PH *et al* Complete surgical metastasectomy of renal cell carcinoma in the post‐cytokine era. J. Urol. 2020; 203: 275–82. 31393812 10.1097/JU.0000000000000488

